# A Result of Vaccination

**Published:** 1894-03

**Authors:** 


### A RESULT OF VACCINATION.

BUCYRUS, O., February 1st.—A pitiable case resulting from vaccination is that of a Bucyrus boy at Waldo, just on the southern edge of the county. Harvey Kenyon was vaccinated three times before it was thought a successful operation had been performed. Young Kenyon is in a horrible condition. His body is black and his feet are literally decaying away, the flesh falling from the bones. The doctors assert that his feet will fall off at the ankles, and that his legs may fall off at the hips before death relieves him. He cannot recover.—*The News and Herald of Cleveland, O., February 2d,* 1894.

